# Serum Phosphate Predicts Early Mortality in Adults Starting Antiretroviral Therapy in Lusaka, Zambia: A Prospective Cohort Study

**DOI:** 10.1371/journal.pone.0010687

**Published:** 2010-05-18

**Authors:** Douglas C. Heimburger, John R. Koethe, Christopher Nyirenda, Claire Bosire, Janelle M. Chiasera, Meridith Blevins, Andres Julian Munoz, Bryan E. Shepherd, Dara Potter, Isaac Zulu, Angela Chisembele-Taylor, Benjamin H. Chi, Jeffrey S. A. Stringer, Edmond K. Kabagambe

**Affiliations:** 1 Centre for Infectious Disease Research in Zambia, Lusaka, Zambia; 2 Institute for Global Health, Vanderbilt University, Nashville, Tennessee, United States of America; 3 Department of Medicine, Vanderbilt University, Nashville, Tennessee, United States of America; 4 Department of Biostatistics, Vanderbilt University, Nashville, Tennessee, United States of America; 5 Department of Internal Medicine, University Teaching Hospital, Lusaka, Zambia; 6 Department of Nutrition Sciences, University of Alabama at Birmingham, Birmingham, Alabama, United States of America; 7 Department of Epidemiology, University of Alabama at Birmingham, Birmingham, Alabama, United States of America; 8 Department of Obstetrics and Gynecology, University of Alabama at Birmingham, Birmingham, Alabama, United States of America; 9 Department of Clinical Laboratory Sciences, University of Alabama at Birmingham, Birmingham, Alabama, United States of America; 10 Department of Internal Medicine, University of Michigan, Ann Arbor, Michigan, United States of America; Institute of Infectious Diseases and Molecular Medicine, South Africa

## Abstract

**Background:**

Patients starting antiretroviral therapy (ART) for acquired immunodeficiency syndrome (AIDS) in sub-Saharan Africa have high rates of mortality in the initial weeks of treatment. We assessed the association of serum phosphate with early mortality among HIV-infected adults with severe malnutrition and/or advanced immunosuppression.

**Methodology/Principal Findings:**

An observational cohort of 142 HIV-infected adults initiating ART in Lusaka, Zambia with body mass index (BMI) <16 kg/m^2^ or CD4^+^ lymphocyte count <50 cells/µL, or both, was followed prospectively during the first 12 weeks of ART. Detailed health and dietary intake history, review of systems, physical examination, serum metabolic panel including phosphate, and serum ferritin and high-sensitivity C-reactive protein (hsCRP) were monitored. The primary outcome was mortality. Baseline serum phosphate was a significant predictor of mortality; participants alive at 12 weeks had a median value of 1.30 mmol/L (interquartile range [IQR]: 1.04, 1.43), compared to 1.06 mmol/L (IQR: 0.89, 1.27) among those who died (p<0.01). Each 0.1 mmol/L increase in baseline phosphate was associated with an incremental decrease in mortality (AHR 0.83; 95% CI 0.72 to 0.95). The association was independent of other metabolic parameters and known risk factors for early ART-associated mortality in sub-Saharan Africa. While participant attrition represented a limitation, it was consistent with local program experience.

**Conclusions/Significance:**

Low serum phosphate at ART initiation was an independent predictor of early mortality among HIV patients starting ART with severe malnutrition or advanced immunosuppression. This may represent a physiologic phenomenon similar to refeeding syndrome, and may lead to therapeutic interventions that could reduce mortality.

## Introduction

Access to antiretroviral therapy (ART) for acquired immunodeficiency syndrome (AIDS) in sub-Saharan Africa has expanded rapidly since 2003, but mortality, especially in the first month after initiating treatment, remains high [1], [2], [3]. The etiology is likely multifactorial, but several analyses from the region have identified low body mass index (BMI) and low CD4^+^ lymphocyte count as powerful predictors of death shortly after starting ART [4], [5], [6], [7].

HIV-associated wasting, defined as a 10% or greater decrease from usual body weight, with concomitant chronic diarrhea or chronic weakness and fever, was recognized as a significant prognostic factor in HIV infection early in the epidemic [8], [9]. In areas where food is scarce, wasting may be accelerated by unbalanced or inadequate energy intake, leading to multiple metabolic abnormalities rarely seen in resource-rich settings. Rapid weight loss or prolonged fasting can deplete serum phosphate stores, leading to a state of hypophosphatemia and the potential for *refeeding syndrome* to develop upon increased nutrient intake [10]. This potentially lethal condition is classically defined by electrolyte and fluid shifts following a reversal from catabolism and fat oxidation to the metabolism of exogenous carbohydrate [11], and predisposes to cardiovascular, respiratory and neurologic sequelae, among others [12], [13]. Rapid improvements in appetite and activity level reported by patients starting ART in sub-Saharan Africa, [14], [15] where dietary staples are often high in carbohydrate (*e.g.,* maize meal and green banana) [16], [17], could conspire to produce a refeeding-like syndrome, especially in patients with severe wasting.

We hypothesized that metabolic abnormalities at the intersection of advanced HIV disease and malnutrition are transiently exacerbated by the introduction of ART and could explain some of the excess early mortality observed in developing countries. Among these could be the refeeding syndrome or a variant of it. We conducted a prospective cohort study to assess the association of serum phosphate levels with early mortality among HIV-infected individuals with severe malnutrition and/or advanced immunosuppression in Lusaka, Zambia.

## Materials and Methods

### Setting

This study enrolled HIV-infected adults initiating ART in a public sector primary care clinic located in the Chawama neighborhood of Lusaka, Zambia. Chawama is a high-density residential area with an estimated population of 56,300 in 2000 [18]. The neighborhood encompasses a range of housing, from planned developments of modern units connected to municipal electricity, water, and sewer systems to crowded, unplanned developments of mud or concrete block structures without electrification. The ART program at Chawama clinic began in March 2006 as part of the Zambian national program for HIV care and treatment [3], [19]. When our study enrollment began in November 2006, 1539 patients were enrolled in care at Chawama clinic and 874 were receiving ART.

### Study outcomes

The primary study endpoint was all-cause mortality in the first 12 weeks of ART. Baseline phosphate concentration was the main exposure variable. Serum phosphate, dietary intake, and calculated energy intake at one week were secondary outcomes, in addition to receipt of phosphate supplementation at any time during the study.

### Eligibility criteria

Individuals were eligible for the study if they qualified for ART according to national guidelines in place at the time (*i.e.,* WHO stage 4 disease, a CD4^+^ lymphocyte count <200 cells/µL, or WHO stage 3 disease and a CD4^+^ lymphocyte count <350 cells/µL) and were intending to start therapy the same day; had a BMI<16 kg/m^2^ or a CD4^+^ lymphocyte count <50 cells/µL; intended to remain in the area for the duration of study; agreed to adhere to the additional study visits and laboratory testing requirements; and agreed to be contacted in the event of a missed study visit.

### Study design and procedures

Individuals who met the study eligibility criteria and provided written informed consent were enrolled on the day of ART initiation. At each study visit, participants were evaluated by a research nurse and a clinical officer (similar to a physician assistant in the US and Europe), and additional evaluation was performed by a supervising physician as needed. The initial visit included a detailed health and 24-hour dietary intake history, review of systems, physical examination, and laboratory testing (serum metabolic panel including phosphate, and serum ferritin and high-sensitivity C-reactive protein (hsCRP)). The first-line ART regimen was selected from the national program formulary: a non-nucleoside reverse transcriptase inhibitor (NNRTI; efavirenz [EFV] or nevirapine [NVP]), in combination with two nucleoside reverse transcriptase inhibitors (NRTIs; lamivudine [3TC] with either zidovudine [ZDV] or stavudine [d4T]). In July 2007, tenofovir with emtricitabine (TDF/FTC) was introduced as the first line NRTI combination by the national program, and study participants enrolled after this time received the new agents [20]. Patients on treatment prior to July 2007 remained on the original regimen, except in cases of treatment failure or toxicity.

Subsequent study visits occurred after 1, 2, 4, 8, and 12 weeks of ART. At each visit, the symptom assessment, physical examination, and metabolic panel were repeated; 24-hour dietary intake recall was repeated after 1, 4, and 12 weeks. Nutrient intake was computed using Nutrition Data System for Research (NDS-R®, Minneapolis, Minnesota, USA; www.ncc.umn.edu). Using the 2005 Zambian Nutrition Commission composition data [21], we matched consumed foods to the NDS-R database food and nutrient content and added recipes of frequently consumed foods to the database. Zambian foods not in the database were substituted with similar foods in the database using recommended tolerance guidelines.

When deficiencies of phosphate were detected, we intervened according to a predetermined algorithm based on serum level. Participants whose levels were 0.65–0.86 mmol/L were counseled to increase their intake of phosphorus-containing foods and provided a World Food Programme macronutrient supplement ration; those whose levels were 0.5–0.64 mmol/L also received oral phosphate supplementation, typically in 7-day allotments, while those with levels <0.5 mmol/L were additionally treated with intravenous phosphate supplementation. Intervention typically occurred at least 7 days after the serum collection due to time required for assay completion and reporting of results to the study clinic.

If a participant missed a study visit, study staff first attempted to contact the individual or a designated representative by mobile phone. If the participant could not be contacted, community outreach teams attempted to locate the patient or a relative using housing locator forms completed at enrollment in HIV care [22]. If the participant could not be located or credible information on vital status could not be obtained from relatives or the community, the participant was classified as lost to follow-up.

The study protocol and informed consent documents were approved by the University of Zambia Research Ethics Committee (Lusaka, Zambia), and the Institutional Review Boards at the University of Alabama at Birmingham (Birmingham, Alabama, USA) and Vanderbilt University (Nashville, Tennessee, USA).

### Laboratory assays

CD4^+^ lymphocyte counts were performed using a Beckman Coulter Epics XL-MCL flow cytometer (Beckman Coulter, Inc., Fullerton, CA, USA), and hemogram or complete blood count with differential using a Horiba ABX Pentra 80 (Horiba ABX Diagnostics Inc., Montpellier, France). Routine and study-specific chemistry assays were measured using a Roche COBAS Integra 400+ (Roche Diagnostics, Basel, Switzerland) or a Pointe 180 Chemistry Analyzer (Pointe Scientific, Canton, MI, USA).

### Statistical methods

We compared differences between survivors and non-survivors with the Wilcoxon rank sum test for continuous variables and the chi-square test for categorical variables. We used Cox proportional hazards regression to determine whether baseline phosphate concentration was associated with mortality and adjusted for potential confounders: sex, age, and baseline hemoglobin [23], [24]. We modeled the relationship between the log-hazard of death and baseline phosphate by first assuming linearity (primary analysis) and then relaxing this assumption using restricted cubic splines with 3 knots. Linearity was assessed using a likelihood ratio test. In the primary analysis, a participant was censored at the last study visit date if vital status at 12 weeks was unknown (*i.e.,* lost to follow-up). A second multivariable regression modeled the relationship between time to death or loss to follow-up and the same predictors.

We accounted for missing values of baseline variables with multiple imputation techniques [25]. In multiple imputation, known baseline participant characteristics are used to predict a series of plausible values for those that are missing. We repeated this process 25 times and performed Cox proportional hazard regression on each imputed data set, producing multiple analysis results. We report the average estimates from these multiple calculations. R-software 2.9.2 (www.r-project.org) was used for data analyses.

## Results

**Figure 1:**
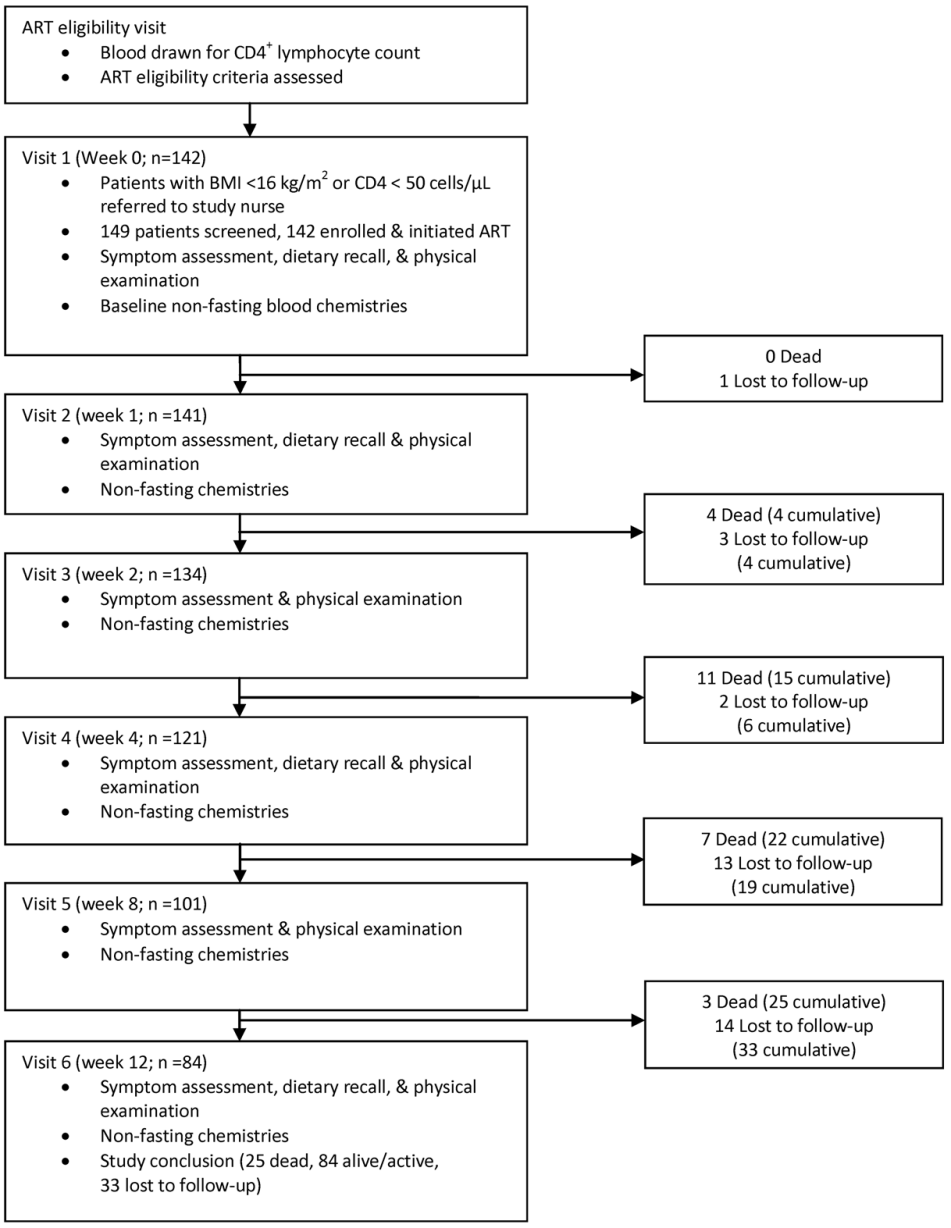
Study procedures and cohort status by study visit.

Between 6 November 2006 and 12 November 2007, 142 participants were enrolled. Figure 1 describes the study visit schedule and the status of the cohort at each visit. Thirty-two (23%) participants met only the BMI criterion for participation, 83 (58%) met only the CD4^+^ lymphocyte criterion, and 27 (19%) met both. Twenty-five participants died over the 12-week follow-up period (mortality rate 87.4 per 100 person-years of follow-up); 10 (40%) participants died within four weeks of starting ART, although none died in the first week. Thirty-three participants (23%) were lost to follow-up; the median follow-up time for those lost was 58 days (interquartile range [IQR]: 45, 71).

Table 1 shows the baseline demographics of the cohort. Participants were predominantly female (61%), consistent with trends seen in the Lusaka HIV care program [3]. The median CD4^+^ lymphocyte count (34 cells/µL) and BMI (16.4 kg/m^2^) were low, with narrow IQRs. There were no clinically meaningful differences between sexes in BMI, CD4^+^ lymphocyte count, hemoglobin, or baseline median serum chemistries (data not shown). First-line ART regimens differed significantly between the sexes: 25% of males received efavirenz-containing regimens compared to 15% of females, while 47% of males received zidovudine-containing regimens compared to 18% of females (p<0.01).

**Table 1:** Baseline participant characteristics.

**N**	142	
**Female, n** (%)	87 (61%)	
**Age**, median years (IQR)	32 (28, 38)	
**Weight**, median kg (IQR)	45.5 (41, 51)	
**BMI**, median kg/m^2^ (IQR)	16.4 (15.4, 18.5)	
**CD4^+^ count**, median cells/µL (IQR)	34 (21, 47)	
**Hemoglobin**, median g/dL (IQR)	9.8 (8.8, 11.6)	
**Serum chemistries**, median (IQR)		**Reference Range ** [**31**]
Phosphate, mmol/L	1.26 (1.03, 1.42)	0.81–1.4
Potassium, mmol/L	4.2 (3.7, 4.6)	3.5–5.0
Chloride, mmol/L	108 (103, 111)	102–109
Bicarbonate, mmol/L	29 (26, 31)	22–30
BUN, mmol/L	3.3 (2.4, 4.7)	2.5–7.1
Creatinine, µmol/L	71 (62, 88)	44–106*
Glucose (non-fasting), mmol/L	3.9 (3.4, 4.8)	4.2–6.1
Magnesium, mmol/L	0.88 (0.79, 0.96)	0.62–0.95
Albumin, g/L	29.5 (23.9, 33.2)	40–53*
Ferritin, µg/L	221 (59, 485)	10–248*
hsCRP, mg/L	2.75 (1.1, 15.2)	0.2–3.0
**ART Regimen, n (%)**		
AZT/3TC/EFV	8 (5.6%)	
AZT/3TC/NVP	34 (23.9%)	
d4T/3TC/EFV	15 (10.6%)	
d4T/3TC/NVP	62 (43.7%)	
TDF/FTC/EFV	4 (2.8%)	
TDF/FTC/NVP	19 (13.4%)	

Abbreviations: ART, antiretroviral therapy; BMI, body mass index; BUN, blood urea nitrogen; hsCRP, high sensitivity C-reactive protein; IQR, interquartile range.

Among the 142 cases included in the multivariable analysis, values were missing for the following measurements: 8 (6%) baseline serum phosphate, 31 (22%) week one serum phosphate, 29 (20%) baseline hemoglobin (because either blood or assay reagents could not be obtained), 2 age (birth dates unknown), and one BMI (participant could not stand for height measurement).

*Normal creatinine range: females 44–80, males 53–106 µmol/L; normal albumin range: females 41–53, males 40–50 g/L; normal ferritin range: females 10–150, males 29–248 µg/L.

We found no significant differences in baseline demographic and medical descriptors, or serum chemistries, between participants alive at 12 weeks versus those lost to follow-up, with the exception of age (Table 2). We also compared participants lost to follow-up with those not lost to follow-up (*i.e.,* known to be alive or dead at 12 weeks) and found no significant differences in baseline descriptors or serum chemistries, with the exception of age (30 versus 34 years; p<0.01) and serum glucose (3.6 versus 4.0 mmol/L; p = 0.03 [data not shown]).

**Table 2:** Baseline participant characteristics by 90-day survival.

	Alive (n = 84)	Dead (n = 25)	p *	Lost (n = 33)	p **
**Female, n** (%)	53 (63%)	12 (48%)	0.26	22 (66.7%)	0.88
**Age**, median years (IQR)	34 (29, 38)	34 (29, 41)	0.72	30 (25, 32)	<0.01
**Weight**, median kg (IQR)	46 (41, 52)	42 (41, 48)	0.20	46 (40, 51)	0.84
**BMI**, median kg/m^2^ (IQR)	16.5 (15.4, 18.6)	16.0 (15.2, 17.0)	0.30	16.7 (15.6, 19.2)	0.48
**CD4^+^ count**, median cells/µL (IQR)	36 (22, 48)	31 (23, 45)	0.94	30 (20, 47)	0.51
**Hemoglobin**, median g/dL (IQR)	10.0 (8.9, 11.7)	9.3 (8.6, 11.1)	0.22	9.7 (8.2, 11.0)	0.20
** Serum chemistries**,	median (IQR)				
Phosphate, mmol/L	1.30 (1.04, 1.43)	1.06 (0.89, 1.27)	<0.01	1.28 (1.18, 1.50)	0.20
Potassium, mmol/L	4.2 (3.7, 4.6)	4.1 (3.5, 4.7)	0.52	4.1 (3.7, 4.5)	0.71
Chloride, mmol/L	108 (103, 110)	103 (97, 112)	0.25	109 (104, 112)	0.33
Bicarbonate, mmol/L	28.8 (25.4, 30.7)	28.1 (26.9, 31.9)	0.44	29.3 (26.5, 30.6)	0.60
BUN, mmol/L	3.3 (2.6, 4.4)	3.6 (2.2, 4.4)	1.00	3.6 (2.1, 4.8)	0.77
Creatinine, µmol/L	75.2 (59.7, 88.4)	88.4 (70.7, 106.1)	0.12	70.7 (61.9, 99.4)	0.86
Glucose, mmol/L	3.9 (3.4, 4.8)	4.8 (3.6, 6.1)	0.07	3.6 (3.3, 4.3)	0.07
Magnesium, mmol/L	0.86 (0.79, 0.97)	0.91 (0.73, 0.98)	0.87	0.89 (0.82, 0.94)	0.61
Albumin, g/L	30.4 (25.8, 33.1)	24.0 (19.9, 29.4)	<0.01	31.4 (22.7, 33.6)	0.65
Ferritin, µg/L	168 (56, 419)	521 (395, 1029)	<0.01	204 (98, 483)	0.42
hsCRP, mg/L	2.4 (1.1, 9.9)	14.5 (4.7, 23)	0.01	2.5 (1.0, 17.0)	0.52
**Week 1 phosphate**, mmol/L (IQR)	1.06 (0.94, 1.26)	1.07 (0.78, 1.16)	0.30	1.08 (0.91, 1.16)	0.86
**Change in baseline to week 1 phosphate**, mmol/L (IQR)	−0.18 (−0.35, 0.01)	−0.08 (−0.17, 0.12)	0.07	−0.14 (−0.55,0.08)	0.83
**% Change in baseline to week 1 phosphate** (IQR)	−14.9% (−26.9, 1.2)	−9.0% (−18.5, 10.9)	0.21	−10.9% (−36.5, 8.9)	0.82
**Reported baseline energy intake**, kcal/d (IQR)	1499 (1028, 1853)	1252 (858, 1724)	0.09	1549 (969, 1851)	0.57
**Reported baseline carbohydrate intake**, g/d (IQR)	203 (153, 283)	181 (123, 237)	0.26	204 (145, 257)	0.51
**Reported week 1 energy intake**, kcal/day (IQR)	1540 (1076, 1762)	939 (726, 1280)	<0.01	1201 (919, 1430)	0.05
**Reported week 1 carbohydrate intake**, g/d (IQR)	207 (151, 261)	151 (113, 196)	<0.01	180 (124, 216)	0.07

Abbreviations: BMI, body mass index; BUN, blood urea nitrogen; hsCRP, high sensitivity C-reactive protein; IQR, interquartile range.

*P-values refer to comparisons between participants dead and alive at 12 weeks.

**P-values refer to comparisons between participants lost to follow-up and alive at 12 weeks. We also compared participants lost to follow-up with those not lost to follow-up (*i.e.,* known to be alive or dead at 12 weeks) and found no significant differences in baseline descriptors or serum chemistries, with the exception of age (30 versus 34 years; p<0.01) and serum glucose (3.6 versus 4.0 mmol/L; p = 0.03) [data not shown].

Baseline serum phosphate was significantly higher among participants alive at 12 weeks (median 1.30 mmol/L; IQR: 1.04, 1.43), compared to those who died (median 1.06 mmol/L; IQR: 0.89, 1.27; p<0.01). Other measured electrolytes were not significantly associated with mortality (Table 2). Overall, 44 (31%) participants had phosphate levels <0.87 mmol/L at some point during the study, and received at least dietary counseling. Seventeen (12%) had levels <0.65 mmol/L at some point, 7 (5%) of which occurred on ≥2 visits despite phosphate supplementation. By week 12, 7 (8%) survivors compared to 5 (20%) deceased participants and 1 (3%) lost participant had received phosphate supplementation orally only (12 persons) or intravenously and orally (1 person) on at least one occasion. One participant met criteria for intravenous supplementation at week 4 (serum phosphate 0.47 mmol/L) but died 8 days later without returning to the clinic. Of those supplemented, 5 (42%) had baseline phosphate levels <0.87 mmol/L; these participants' median (IQR) time to supplementation was 14 (13, 21) days and their median (IQR) duration on supplementation was 7 (7, 7) days. Seven (58%) supplemented participants had baseline phosphate levels ≥0.87 mmol/L but required supplementation after subsequent visits. These participants' median (IQR) time to supplementation was 29 (20.5, 61) days and their median (IQR) duration on supplementation was 6 (6, 7) days. Mortality did not statistically differ by phosphate supplementation (p = 0.20).

At baseline there were no significant differences in appetite or dietary intake of survivors compared to non-survivors (p>0.05). However, after one week of ART, appetite and intakes of carbohydrate and total energy were significantly lower in non-survivors than in survivors (p<0.01).

After adjusting for sex, age, CD4^+^ lymphocyte count, BMI, and baseline hemoglobin, low baseline serum phosphate was associated with an increased risk of death within 12 weeks of ART initiation (p = 0.008, Table 3). For each 0.1 mmol/L increase in baseline phosphate, the estimated hazard of death decreased 17% (adjusted hazard ratio (AHR) 0.83; 95% CI 0.72 to 0.95). BMI, CD4^+^ lymphocyte count, and hemoglobin were not significant predictors of mortality in this model. In secondary analyses that adjusted for significant variables in Table 2 (*i.e.,* albumin, ferritin, and hsCRP) baseline phosphate remained a significant predictor of mortality (p<0.05; data not shown).

**Table 3:** Adjusted hazard ratios for mortality at 12 weeks (baseline variables).

	AHR (95% CI)*	p-value
**Male sex**	1.56 (0.64 to 3.84)	0.33
**Age** (per 10 years)	1.17 (0.66 to 2.08)	0.59
**CD4^+^ count** (per 10 cells/µL increase)	1.02 (0.90 to 1.15)	0.74
**BMI** (per 1 kg/m^2^ increase)	0.83 (0.65 to 1.06)	0.14
**Hemoglobin** (per 1 g/dL increase)	0.87 (0.70 to 1.08)	0.21
**Serum phosphate** (per 0.1 mmol/L increase)	0.83 (0.72 to 0.95)	0.008

There is little evidence that the association between phosphate and the hazard of death is non-linear based on a likelihood ratio test (p = 0.27). Similarly, there is little evidence that the association between any continuous variable and the hazard of death is non-linear (p>0.20 for each).

There was insufficient evidence to conclude that the relationship between baseline serum phosphate and the log-hazard of death was non-linear (p = 0.27), although baseline phosphate remained a good predictor of mortality in secondary analyses that relaxed the linearity assumption (p = 0.05; data not shown). Baseline phosphate was not associated with an increased risk of death or loss to follow-up (AHR = 0.98 compared to patients who were active and alive, 95% CI 0.90 to 1.06). While the absolute phosphate change from baseline to week one trended toward statistical significance (p = 0.07), the percentage decrease (14.9% [alive] versus 9.0% [deceased]) did not (p = 0.21). Additionally, median week one phosphate values did not differ significantly between alive (1.06) and subsequently deceased (1.07) participants.

## Discussion

In this observational cohort of HIV-infected persons with severe malnutrition, advanced immunosuppression, or both, a low serum phosphate at ART initiation predicted lower survival probability at 12 weeks. The increased hazard of death conferred by low serum phosphate was independent of other metabolic parameters, hsCRP, and known risk factors for early ART-associated mortality in sub-Saharan Africa, including low BMI, CD4^+^ lymphocyte count and hemoglobin.

Our findings may represent a variant of the refeeding syndrome associated with advanced HIV disease, in which low pre-ART serum phosphate is insufficient to maintain homeostasis in response to metabolic changes accompanying treatment initiation. The increased appetite and capacity for physical activity often reported by patients starting ART could reflect a burst of phosphate-dependent cellular respiration and ATP generation in response to reduced systemic inflammation and HIV-1 viral replication [14], [15]. This is but one speculative hypothesis for our findings, and further research into the metabolic consequences of ART in advanced HIV infection is warranted. The lower appetite and dietary intake among non-survivors after one week of ART may indicate overall worsening of their condition and suggests that the phosphate-mortality interaction did not strictly represent refeeding.

The lack of association between week one phosphate and subsequent mortality suggests that acute electrolyte derangements were not a proximate cause of death in most instances. However, 10 deaths (40%) occurred in the second through fourth weeks of ART. This timing is consistent with reports of refeeding syndrome deaths among starving prisoners liberated from internment after World War II [26], [27], but it could also be consistent with immune reconstitution inflammatory syndrome (IRIS). At least one study participant exhibited findings consistent with classic refeeding syndrome: a 34-year-old male with severe hypophosphatemia developed anasarca after 6 weeks of ART despite oral and intravenous phosphorus supplementation. It resolved after 2 weeks of further supplementation and administration of a low-dose diuretic [28].

Baseline serum phosphate retained significance as a predictor of survival independent of renal function, acid-base status, albumin, ferritin and hsCRP. Significantly higher baseline levels of ferritin and hsCRP, both markers of systemic inflammation, among non-survivors may indicate a greater burden of undiagnosed opportunistic infections or an exaggerated response to HIV-1 viremia. The presence or absence of occult co-infections could not be adequately explored in this study because of limited diagnostic capabilities in the study setting, which was representative of public-sector clinics in Lusaka Zambia. Further prospective studies to characterize the etiology and epidemiology of occult infections in severely immunocompromised HIV-infected adults using advanced diagnostic modalities are needed to explore this.

Thirteen participants received phosphate supplementation because of low serum levels. While supplementation was not significantly associated with survival, we cannot conclude that it had no effect without an appropriate comparison group. Rather, because we were ethically obligated to provide supplementation to all participants with low phosphate levels, supplementation was essentially a marker for hypophosphatemia. To the extent that supplementation may have reduced mortality, it would bias our results toward the null hypothesis of observing no mortality difference between those with low serum phosphate and those with normal phosphate.

The observational design of our study prevents us from concluding a causal relationship between low serum phosphate and mortality. The study was also limited by participant attrition, but the number of lost participants was not atypical. The observed loss rates are similar to reports from other programmatic cohorts in sub-Saharan Africa, and previous analyses of the Zambian ART program have reported a similar lost to follow-up rate prior to 6 months among low BMI patients [29], [30]. Except for slight differences in median age and serum glucose, those who were lost to follow-up did not differ substantially when compared to either survivors or all participants not lost to follow-up.

Relative homogeneity of known markers of advanced HIV among study participants, indicated by narrow interquartile ranges (Table 1), likely contributed to the finding that BMI, CD4+ count, and hemoglobin were not significant predictors of mortality. The independence of baseline serum phosphate as a predictor of mortality is bolstered by our study inclusion criteria, which limited the potential impact of known confounders.

In this observational cohort study, serum phosphate at ART initiation was an independent predictor of mortality among patients with advanced HIV disease. This may represent a physiologic phenomenon similar to the refeeding syndrome. A randomized prospective trial of phosphate repletion among patients with low BMIs or low serum levels, either through electrolyte or nutritional supplementation, is warranted to address causality and explore the therapeutic implications of these findings.
